# Cardiac Alpha-Myosin (*MYH6*) Is the Predominant Sarcomeric Disease Gene for Familial Atrial Septal Defects

**DOI:** 10.1371/journal.pone.0028872

**Published:** 2011-12-14

**Authors:** Maximilian G. Posch, Stephan Waldmuller, Melanie Müller, Thomas Scheffold, David Fournier, Miguel A. Andrade-Navarro, Bernard De Geeter, Sophie Guillaumont, Claire Dauphin, Dany Yousseff, Katharina R. Schmitt, Andreas Perrot, Felix Berger, Roland Hetzer, Patrice Bouvagnet, Cemil Özcelik

**Affiliations:** 1 Department of Cardiothoracic and Vascular Surgery, Deutsches Herzzentrum Berlin, Berlin, Germany; 2 Experimental and Clinical Research Center (ECRC), Charité - Universitätsmedizin Berlin, Berlin, Germany; 3 Institute for Heart and Circulation Research, Dortmund, Germany; 4 Computational Biology and Data Mining Group, Max-Delbrück Center for Molecular Medicine, Berlin, Germany; 5 Service de Cardiologie, Clinique Sainte Odile, Strasbourg, France; 6 Institut Marin Saint Pierre, Palavas Les Flots, France; 7 Centre Hospitalier Gabriel Montpied, Clermont-Ferrand, France; 8 Hôpital d'Enfants, Vandoeuvre les Nancy, France; 9 Department for Congenital Heart Disease/Pediatric Cardiology, Deutsches Herzzentrum Berlin, Berlin, Germany; 10 Laboratoire Cardiogénétique, Hospices Civils de Lyon, Lyon, France; National Cancer Center, Japan

## Abstract

Secundum-type atrial septal defects (ASDII) account for approximately 10% of all congenital heart defects (CHD) and are associated with a familial risk. Mutations in transcription factors represent a genetic source for ASDII. Yet, little is known about the role of mutations in sarcomeric genes in ASDII etiology. To assess the role of sarcomeric genes in patients with inherited ASDII, we analyzed 13 sarcomeric genes (*MYH7*, *MYBPC3*, *TNNT2*, *TCAP*, *TNNI3*, *MYH6*, *TPM1*, *MYL2*, *CSRP3*, *ACTC1*, *MYL3*, *TNNC1*, and *TTN* kinase region) in 31 patients with familial ASDII using array-based resequencing. Genotyping of family relatives and control subjects as well as structural and homology analyses were used to evaluate the pathogenic impact of novel non-synonymous gene variants. Three novel missense mutations were found in the *MYH6* gene encoding alpha-myosin heavy chain (*R17H*, *C539R*, and *K543R*). These mutations co-segregated with CHD in the families and were absent in 370 control alleles. Interestingly, all three *MYH6* mutations are located in a highly conserved region of the alpha-myosin motor domain, which is involved in myosin-actin interaction. In addition, the cardiomyopathy related *MYH6*-A1004S and the *MYBPC3*-A833T mutations were also found in one and two unrelated subjects with ASDII, respectively. No mutations were found in the 11 other sarcomeric genes analyzed. The study indicates that sarcomeric gene mutations may represent a so far underestimated genetic source for familial recurrence of ASDII. In particular, perturbations in the *MYH6* head domain seem to play a major role in the genetic origin of familial ASDII.

## Introduction

Congenital heart defects (CHD) are the most common inborn malformations and are a major cause of perinatal mortality. Though the etiology of CHD is largely unknown, epidemiological studies indicate a genetic component [1]. The ostium secundum atrial septal defect (ASDII) accounts for 10% of all CHD and shows familial recurrence in approximately 8% [2]. In recent years, single gene mutations were identified as heritable risk factors for familial ASDII [3]. Though mutations in cardiac transcription factors are believed to represent a major genetic source for ASDII, mutations in non-regulatory genes were also found [3]. In patients with familial ASDII, pedigree based linkage analyses identified mutations in *MYH6* and *ACTC1*, which encode the sarcomeric filament proteins alpha-myosin heavy chain and cardiac alpha-actin, respectively [4], [5]. Sarcomeric genes are well recognized as disease genes for diverse familial cardiomyopathies [6]. So far, more than ten different sarcomeric genes have been classified as cardiomyopathy disease genes. A subset of patients with familial dilated cardiomyopathy (DCM) harbors additional cardiac anomalies including ASDII [7]. ASDII was also reported to be overrepresented in patients with hypertrophic cardiomyopathy (HCM) or left-ventricular non-compaction cardiomyopathy (LVNC) caused by mutations in *ACTC1* and *MYH7* encoding the sarcomeric filaments alpha-actin and beta-myosin heavy chain, respectively [8], [9]. Moreover, recent studies have reported mutations in *MYH6* in patients with various forms of CHD [10] and *MYH7* mutations in congenital Ebstein anomaly in combination with LVNC [11].

To systematically investigate the role of sarcomeric cardiomyopathy disease genes as a genetic source for ASDII, we analyzed the coding region of 13 sarcomeric genes using array-based resequencing technology in 31 patients with proven familial ASDII. In the present study, we provide further evidence for a connection between CHD and the sarcomere, especially the thick filamentous alpha-myosin heavy chain.

## Results

Thirty-one patients with proven familial ASDII were investigated. The number of family members affected by CHD ranged from 2 to 12 (mean 3.7). Mutations in *NKX2.5* and *GATA4* were previously excluded in all probands. In total, 205 sequence variations including 134 intronic and 71 exonic (of these 13 non-synonymous and 58 synonymous) variations were found among 16 genes (Supporting data S1). These comprised mostly known single nucleotide polymorphisms (SNPs) registered in DB SNP and or exome variant server (EVS) [12]. After careful bioinformatic analysis we extracted five distinct rare mutations (four *MYH6* and one *MYBPC3* mutations) in six unrelated ASDII index patients. All mutations were heterozygous missense mutations. Three *MYH6* mutations were novel and two (one *MYH6* and one *MYBPC3*) were previously found in patients with familial cardiomyopathies. The mutations were absent in 370 control alleles from ethnically matched individuals without CHD. Moreover, the three novel *MYH6* mutations were not found among more than 4,800 European or African American individuals according to EVS. All available family members were clinically characterized and genotyped. The phenotypes of all MYH6 mutation carriers are given in table 1.

**Table 1:** Phenotype of family members with *MYH6* mutations.

Pedigree	ID	Nucleotide change	Amino acid change	Phenotype
MC081	II:2	c.50G>A	R17H	Normal
	III:1	c.50G>A	R17H	AVSD, left SVC to CS
	**III:2**	**c.50G>A**	**R17H**	**ASD**
	III:3	c.50G>A	R17H	AVSD, left SVC to CS
MC027	I:1	c.1615T>C	C539R	ASD
	II:2	c.1615T>C	C539R	ASD
	**III:1**	**c.1615T>C**	**C539R**	**ASD**
MC053	I:1	c.1628A>G	K543R	Normal
	**II:2**	**c.1628A>G**	**K543R**	**ASD**
	II:3	c.1628A>G	K543R	n.a.
	III:3	c.1628A>G	K543R	ASD
MC078	I:1	c.3010G>T	A1004S	n.a. (heart murmur)
	II:2	c.3010G>T	A1004S	Normal
	II:3	c.3010G>T	A1004S	ASD
	II:4	c.3010G>T	A1004S	Normal
	II:6	c.3010G>T	A1004S	Normal
	**II:8**	**c.3010G>T**	**A1004S**	**ASD**

AVSD – atrioventricular septal defect; SVC - superior vena cava; CS – coronary sinus; n.a.- not assessed; The index patients are highlighted in bold letters.

### Alpha-myosin heavy chain (*MYH6*)

#### Family MC081 (Figure 1A)

**Figure 1:**
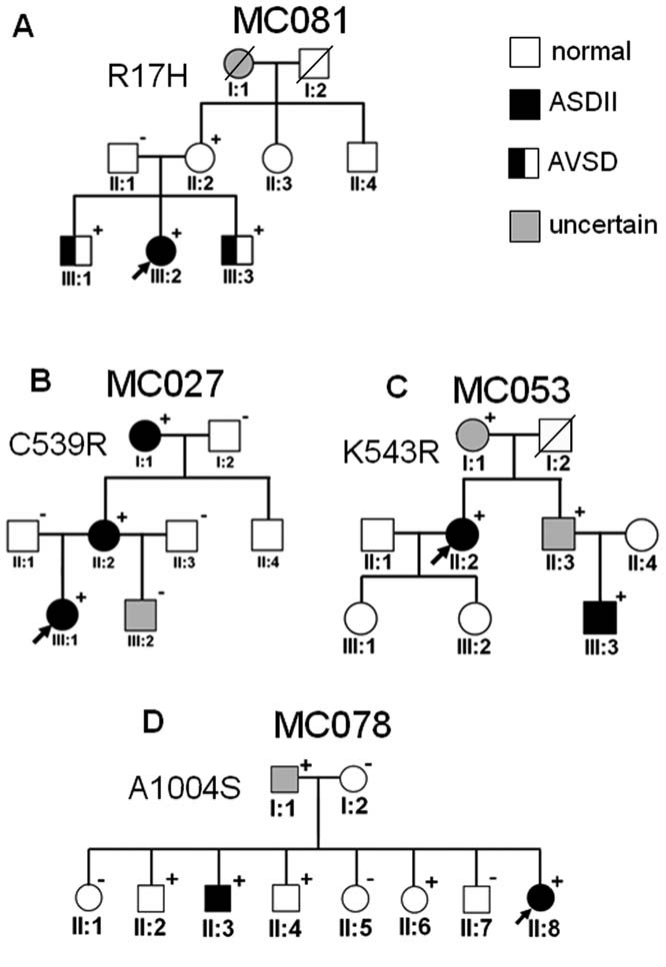
Family pedigrees of *MYH6* mutation carriers. Squares represent male and circles female subjects. The proband is marked by an arrow. Plus signs and minus signs depict mutation-positive and mutation-negative individuals, respectively. Crossed symbols represent deceased individuals.

We found a heterozygous G>A transition at position c.50 in exon 2 of *MYH6* resulting in a replacement of arginine by histidine in the N-terminal head domain of *MYH6* (R17H). The carrier (III:2) was a 10-year-old girl with ASDII, which closed spontaneously during early childhood. Her elder brother (III:1) was also harboring R17H and was affected by an atrioventricular septal defect (AVSD), with a left superior vena cava draining into the coronary sinus. He underwent surgical AV canal closure at age of 4 years. The mutation was also present in the younger brother (III:3) who had a cardiac phenotype similar to that of his brother (complete AV canal and anomalous draining of a left superior vena cava into the coronary sinus). He had to undergo cardiac surgery within the first year after birth. Prenatal genetic testing revealed a normal karyotype in III:1 and III:3. There were no apparent syndromic stigmata among the three siblings. The mutation was transmitted by their mother who did not demonstrate any cardiac anomaly. The maternal grandmother was reported to have a mitral valve defect requiring cardiac surgery. The maternal grandfather died from silicosis. The mutation affects residue R17, which is in the highly conserved N-terminal region of a subfamily of class II myosin heavy chains but not in class II myosin heavy chains *MYH9, MYH10*, *MYH11* and *MYH4* or in other myosin heavy chains (see Supporting data S2A and S4). This residue is conserved in chicken myosin-VI and included in a solved structure (PDB code 2MYS) [13]; although the corresponding part of the structure is not very well resolved it appears to be fully buried and mutation to a bulky residue such as histidine would probably disrupt the structure.

#### Family MC027 (Figure 1B)

A heterozygous T>C transition at position c.1615 in exon 14 of MYH6, which replaces cysteine by arginine at residue 539 (C539R) was found in a 6-year-old girl with ASDII (III:1). The mutation was inherited from her mother (II:2) who also had ASDII requiring surgical closure at age of 34 years. The maternal grandmother (I:1) was also harboring C539R and had ASDII, which was surgically closed at age of 50 years. C539 lies within the loop of a helix-loop-helix motif that is crucially involved in the myosin-actin interaction [13]. The C539R mutation is located at the start of the loop and is conserved in human myosin heavy chains class II, I, V, and XV. It is a serine in myosin heavy chains class III, VII, IX and X, an asparagine in myosin heavy chains class VI, and an alanine or valine in myosin heavy chains class XVIII (see Supporting data S2B).

#### Family MC053 (Figure 1C)

Another mutation in this region was a heterozygous A>G transition at nucleotide c.1628 in exon 14 resulting in a replacement of lysine by arginine at residue 543 (K543R). The carrier was a 58-year-old female (II:2) with ASDII. The mutation was inherited from her mother whose disease status remains unclear since she refused clinical workup. The father died from lung cancer. The nephew (III:3) was also diagnosed with ASDII when aged four years and harbored K543R. Similar to the C539R mutation, the K543R mutation also affects the highly conserved alpha myosin helix-loop-helix motif. The residue is highly conserved in myosin heavy chain class II, and is substituted heterogeneously in other classes, glycine, lysine and glutamine being the most frequent residues at this position (Supporting data S2B).

#### Family MC078 (Figure 1D)

A heterozygous G>T transversion in exon 22 was found at codon c.3010 of *MYH6* (A1004S) in a four-year-old girl (II:8) with cribriform ASDII. The mutation was previously described in patients with DCM [14], [15]. Among seven elder siblings, one brother (II:3) harbouring A1004S had ASDII, which was surgically closed at age of 9 years. Three further siblings (II:2, II:4, II:6) were also positive whereas three others (II:1, II:5, II:7) were negative for A1004S. None of them revealed CHD. The mutation was inherited from their father. Yet, he did not undergo echocardiography to evaluate his cardiac phenotype. The mutation lies in the neck domain of alpha myosin heavy chain, and is located in a portion of sequence predicted as alpha-helicoidal according to Jpred3 [16]. The affected residue is conserved in a subfamily of class II myosin heavy chains that groups with *MYH6* except for *MYH8*, which has a threonine instead (Supporting data S2C), and it is an arginine in the other branch of class II myosin heavy chains (*MYH9*, *MYH10*, *MYH11* and *MYH4*; Supporting data S2C). This region has no obvious sequence similarity to other myosin heavy chains.

### Myosin-binding protein C (*MYBPC3*)

The A>G transition at position c.2497 in exon 26 of MYBPC3 (A833T) was found in two unrelated subjects with ASDII. The mutation was previously identified in patients with HCM and DCM [17], [18], [19]. In the present study, a 49-year-old female with ASDII was heterozygous for A833T. Her son was also affected by ASDII and her grandson was diagnosed with DCM at the age of 6 years. However, family members declined genetic evaluation. The A833T mutation was also found in a 28-year-old male with ASDII who underwent interventional closure of the defect. In addition his father was affected by ASDII, which was also closed interventionally when he was 59 years of age. However, he was negative for the mutation, indicating that the condition of this family may not be (or at least not solely) due to the mutation.

### Novel sarcomeric gene variants of uncertain pathogenicity and novel benign variants

In addition to the aberrations described above, we detected a heterozygous intronic T>C variation in the *MYH6* region at position −2 relative to the start of the gene (GenBank record for *Homo sapiens* chromosome 14 genomic contig, GRCh37.p2 [NT_026437]). The variation was found in a 59-year-old male with ASDII and atrial septal aneurysm. The variant was also found in his mother, who was affected by ASDII, but not in his daughter who also had ASDII. Since the effect on the transcription of the protein remains unclear, we classified it as a variant of uncertain pathogenicity. Further, two novel synonymous variants were found in the *MYH6* gene (A1203A and I1209I) in two Caucasian ASDII patients. According to the ESV database [12] these variations are absent in more than 2,000 European Americans but represent polymorphism among African Americans with a prevalence of 1.8% and 2.2%, respectively (see supporting data S2). Neither novel variations nor known cardiomyopathy-related mutations were found in *MYH7*, *TNNT2*, *TNNI3*, *TNNC1*, *ACTC1*, *MYL2*, *MYL3*, *CSRP3*, *TCAP*, *TPM1* or in the *TTN* kinase region.

## Discussion

We employed an established array based sequence analysis tool developed for genetic diagnosis of familial HCM. As reported previously, the accuracy of the HCM1 array exceeds 99% and is comparable to that of capillary sequencing [20]. Similar performances were also found for the HCM2 array (unpublished data). Although array-based resequencing increases the number of genes that can be analyzed simultaneously, more efficient high-throughput DNA sequencing technologies have emerged during the last years commonly referred to as next generation sequencing (NGS). These technologies are based on massive parallel sequencing of millions of DNA templates through cycles of enzymatic treatment and image-based data acquisition [21]. Although the dissemination of NGS into the clinical diagnostic realm is in its early stages, first approaches in genetic diagnostics of cardiac diseases by NGS seem quite promising [22] and could also be used in the future for cases with atrial septal defects in order to identify novel genes. In the present study we were only able to analyze the coding regions of 13 sarcomeric genes that are covered by the two arrays. However, we believe it is a sufficient strategy to investigate the hypothesis if sarcomeric gene mutations are related to human ASDII. Thirty-one carefully selected probands with proven familial ASDII from two clinical referral centers were included in the study after exclusion of mutations in *NKX2.5* and *GATA4* [23].

**Figure 2:**
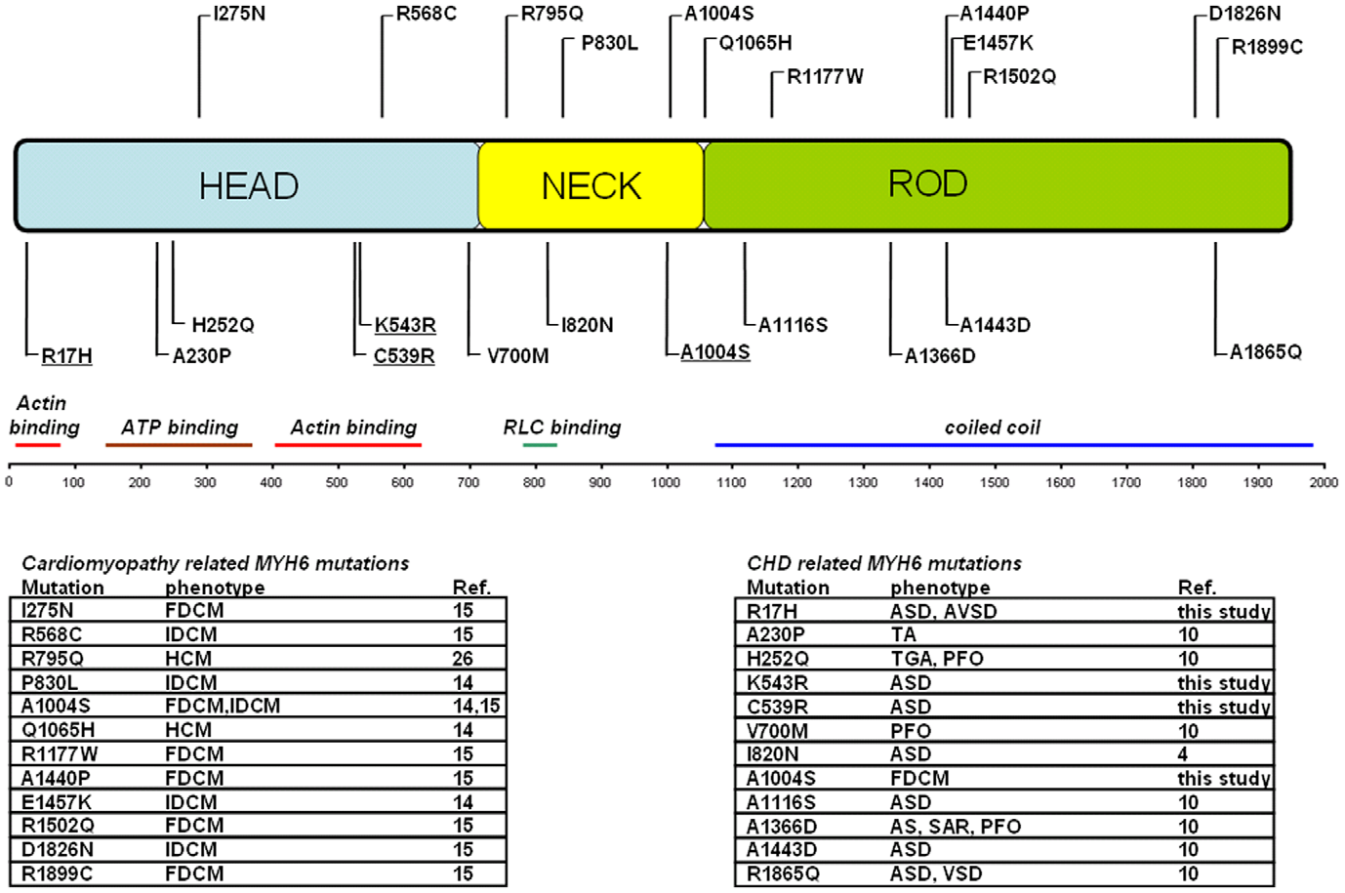
Localization of all so far detected *MYH6* mutations. Centrally shown are the three main domains of alpha-myosin heavy chain (head, neck, and rod). Identified mutations in patients with cardiomyopathy are shown above the protein while mutations in patients with congenital heart disease (CHD) are shown below. Mutations found in this study are underlined. The interaction sites for alpha-actin, adenosine triphosphate (ATP), and regulatory myosin light chain (RLC) as well as the known coiled coil structure are indicated. The human cardiac phenotypes to each mutation are given in the table below. FDCM: familial DCM; IDCM: idiopathic DCM; TA: tricuspid atresia; TGA: transposition of the great arteries; PFO: patent foramen ovale; SAR: subaortic rim; AS: Aortic stenosis.

In four out of 31 probands (13%) mutations in *MYH6* were identified, whereas no mutations were found in 11 other sarcomeric genes. *MYH6* encodes alpha-myosin heavy chain expressed in the developing atria [24]. Rutland et al. showed that knockdown of *MYH6* in chick embryos resulted in inhibition of atrial septal morphogenesis [25]. Subsequent structural analysis indicated that the interaction of normal alpha-actin and mutated alpha-myosin may perturb sarcomere formation. Therefore, the authors proposed that disturbed actin-myosin assembly may lead to a failure of atrial septal morphogenesis [25]. Interestingly, in the present study, three out of four ASDII-related *MYH6* mutations are located in the head domain of alpha-myosin responsible for actin-binding. In contrast, mutational analyses of *MYH6* in probands with cardiomyopathy found that the majority of mutations were located in the myosin rod or neck [14], [15], [26] whereas only very few were detected in the head (Figure 2) [15]. In a recent study, Granados-Riveron and colleagues found three out of four *MYH6* mutations in the rod domain mutations among 55 probands with ASDII. Five additional MYH6 mutations were identified among 415 subjects with diverse CHD [10]. Therefore, one may assume that MYH6 mutations are most frequently present in ASDII patients than in subjects with other CHD, with a substantial fraction located in the head domain (Figure 2).

We used control genotyping, cosegregation analysis, sequence homology assessment, and structure analyses to evaluate the potential pathogenic impact of the *MYH6* mutations. In addition all potential mutations were checked in the EVS database [12]. Although the mutations mostly cosegregated with CHD in the families, we also observed the common CHD features of decreased penetrance and variability of expressivity. In family MC081, the R17H mutation seems to be associated with a dominant trait and incomplete disease penetrance. The types of observed cardiac malformations were ASDII and non-syndromic AVSD – a CHD which is mainly attributed to defects of endocardial cushion formation. To our knowledge, AVSD had never been previously associated with sarcomeric gene mutations. In the study by Granados-Riveron et al., no *MYH6* mutations were found in 10 patients with AVSD [10]. On the molecular level the replacement of arginine by histidine maintains a positively charged residue but only arginine (and not histidine) can establish hydrogen bonds to negatively charged residues. The R17H mutation was predicted to be “probably damaging” by the PolyPhen-2 software tool for prediction of effects of missense mutations [27] and was not found among 4,800 American individuals of European or African descent [12]. The C539R and K543R mutations are both located right in the middle loop of the helix-loop-helix that represents a major participant in the interface of actin-myosin according to Holmes et al. [13] Whereas all probands carrying C539R had ASDII, the K543R mutation seems to be associated with incomplete disease penetrance. Both mutations are very likely to modify the interaction of alpha-myosin with actin according to the model of their interaction [13]. The C539R mutation results in a much longer side chain, which likely collides with a neighboring helix and may produce an opening of the helix-loop-helix according to the structures of either the chicken myosin-V (39% identical; supporting data S5a) or the ortholog chicken myosin-VI (81% identical, not shown). The K543R mutation results in the substitution of a side chain for a slightly bulkier one pointing in the direction of actin, according to the definition of the myosin-actin interface [13], and therefore may compromise the interaction (supporting data S5b). Whereas C539R was predicted to be probably damaging, the K543R mutation was predicted as benign according to the PolyPhen-2 tool. Yet, both mutations were excluded in 185 controls in the present study and among 4,800 individuals according to EVS [12]. The A1004S mutation was previously identified in three patients with sporadic DCM and one patient with familial DCM [14], [15]. In the present study, we could not find evidence for DCM in any patient harboring A1004S. Yet a significant portion of A1004 carriers had a normal cardiac phenotype. This mutation is the only one located in the myosin neck, which is predicted to be mostly alpha-helicoidal but whose detailed structure is so far unknown. A 3D structure model for the chicken homolog *MYH11* based on a low resolution image from cryomicroscopy covers up to the first 100 amino acids of the myosin rod suggesting two long homodimerizing alpha helices [28] but the region homologous to A1004 is not part of the model. Nevertheless, this model and a previous study show that the integrity of the myosin rod is important for homodimerization and regulation myosin [29]. The mutations in this region may therefore impair the homodimerization properties of *MYH6*. However, the fact that the A1004S mutation was now found in four different families with different cardiac diseases in addition to healthy family relatives raises some doubt about the true pathogenicity of this variation. According to EVS the A1004S variation has a frequency of slightly more than 0.1% in Europeans (see supporting data S1). Even less certain is the role of the *MYBPC3*-A833T variant, which was found in different studies revealing incomplete familial segregation with HCM and DCM [17], [18], [19]. Although we found a grandson of the proband with ASDII to be affected by cardiomyopathy, we were not able to investigate his genetic status. The A833T variation is present in 0.3% of European Americans according to EVS [12].

A number of *MYH6* mutations are related to myocardial diseases as shown in Figure 2. Obviously, effects of these mutations can manifest during development, causing congenital heart malformations, whereas others cause cardiomyopathy during postnatal life. But the mechanisms underlying this expression diversity still have to be elucidated. Interestingly, mutations in the other main cardiac myosin heavy chain isoform, beta-myosin (*MYH7*), can also lead to phenotypically distinct diseases [8], [11], [30]. Besides familial HCM, DCM and LVNC, Laing early onset distal myopathy and myosin storage myopathy can be caused by *MYH7* mutations. However, no such mutations could be found in ASDII patients, which was confirmed by the present study. Expression differences of the two myosin isoforms in cardiac muscle may provide a hint why only *MYH6* is associated with atrial septal defects. As shown in earlier studies, alpha-myosin is expressed in human atria but not in ventricles whereas beta-myosin is expressed exclusively in the ventricles [24], [31]. To conclude, in the present study we identified three ASDII related MYH6 mutations that were not reported before. Interestingly, all mutations are localized in the motor region of alpha-myosin involved in myosin actin interaction. Our study confirms the assumption that ASDII lies within the diverse spectrum of structural heart anomalies that may be related to sarcomeric gene defects in an autosomal dominant manner. Among various sarcomeric cardiomyopathy disease genes the alpha-myosin heavy chain seems to play a major role in ASDII etiology.

## Materials and Methods

### Patients and clinical evaluation

Thirty-one ASDII patients with a positive family history (at least one relative with ASDII or other congenital septation defect) were included in the study. All patients were of European (Caucasian) origin including ancestors from France, Germany, Spain and Poland. Patients were attending either the Department of Congenital Heart Disease/Pediatric Cardiology at the Deutsches Herzzentrum Berlin (German Heart Institute Berlin), Germany, or were part of the French National Registry of Familial Congenital Heart Defects based at the Groupe Hospitalier Est in Lyon, France. All patients (or legal guardians of minors) gave written informed consent after approval of the institutional review board of the Charite - Universitätsmedizin Berlin. One hundred eighty five Caucasian (and therefore ethnically matched) control patients were selected; 96 of them underwent transesophageal echocardiography to exclude atrial shunts.

### Genetic analysis

Array-based resequencing (ABR) was used to analyze all exonic regions and flanking intronic sequences of 16 genes. Two different Affymetrix (High Wycombe, UK) arrays were deployed covering a total of 13 sarcomeric and three non-sarcomeric hypertrophic cardiomyopathy (HCM) disease genes: the first array (referred to as the “HCM1 array”) covers the three major HCM disease genes *MYH7*, *MYBPC3*, and *TNNT2*. ABR using the HCM1 array was conducted as described [20], with two exceptions where LongAmp® Taq polymerase was used for DNA amplification, and SeqC software (JSi Medical Systems, Kippenheim, Germany) was used subsequently to GSEQ 4.1 software (Affymetrix) to detect mutations. Essentially the same resequencing protocol was used for the second array (referred to as the “HCM2 array”) that was designed to analyze ten sarcomeric and sarcomere-associated genes (*TCAP*, *TNNI3*, *MYH6*, *TPM1*, *MYL2*, *CSRP3*, *ACTC1*, *MYL3*, *TNNC1*, *TTN* kinase region) and three non-sarcomeric genes (*GLA*, *CRYAB*, *PRKAG2*). All possibly disease-causing mutations were confirmed in the index patient (and followed up in relatives) by Sanger sequencing following standard protocols. Details on the primers and protocols used are available upon request.

### Homology analysis of human myosin heavy chain proteins and homologs of solved structure

We found that the orthologs of human MYH6 were not useful for the analysis of residue conservation since they are not divergent enough from the human sequence with which they have identity percentages ranging from 83% of *Danio rerio* MYH6 to 97% of murine MYH6 when comparing full length sequences. Accordingly, the multiple sequence alignment of these sequences indicated that all mutations described here were fully conserved (data not shown). To gain a better idea of residue conservation we retrieved from the Entrez gene database the records of the 37 human genes for myosin heavy chains plus four homologs of solved structure (Supporting data S3). One human gene is a pseudogene (*MYH16*). The other genes coded for protein products whose sequences were obtained from the Entrez protein database and aligned using the MUSCLE algorithm [32] via the EBI server (Supporting data S2). A phylogenetic tree of the resulting alignment was obtained using ClustalX [33] and represented with NJplot [34] (Supporting data S4), indicating good agreement of the alignment with the known myosin classes.

### Structural representation of *MYH6* mutants

Since we were interested in the assessment of the molecular effect of the *MYH6* mutants we represented them using the structure of the myosin head domain of chicken myosin-V (PDB code 1OE9) [35]. These sequences are 39% identical over their myosin head domains and therefore very likely to have similar structures. Moreover, after alignment we observed that the chicken protein shares the residues, the object of this study that lie in the myosin head domain. These residues were highlighted in the corresponding structure and the effects of mutations were studied simply by substituting the corresponding side chains keeping their original direction (see Supporting data S5). Structures were displayed and manipulated using the PyMOL Molecular Graphics System software (DeLano Scientific, Palo Alto, California).

## Supporting Information

Data S1 Gene variants identified in 31 index patients with familial ASDII.
(PDF)

Data S2 Fragments of a multiple sequence alignment of all 37 human myosin heavy chains with four homologs of known structure. Sequence identifiers and other sequence information are available in Supporting data 1. The yellow and green blocks represent alpha-helix and beta-sheet in solved structures. (A) Region around R17. (B) Region around C539 and K543. (C) Region around A1004.
(PDF)

Data S3 Database record identifiers and annotations of the 37 human genes for myosin heavy chains and of four homologs of solved structure.
(PDF)

Data S4 Phylogenetic tree of the sequences aligned in the supporting data 1. Classes are indicated.
(PDF)

Data S5 Zoomed view of the structure from chicken myosin V (PDB:1OE9) [35]. The structure is displayed as a ribbon with the helix-loop-helix colored in red. (A) Original residues equivalent to human *MYH6*-K543 and *MYH6*-C539 are highlighted with side chains represented as sticks. These residues are conserved in the structure represented. (B) For illustration, mutants are represented by simple substitution of side chains without modifying the structure. R539 collides with the nearby helix since it is bulkier than the wild type residue. R543, also with a larger side chain than that of the wild type residue, points to the surface of interaction with actin according to a model by Holmes et al. [13]. This substitution may compromise the interaction between actin and myosin heavy chain.
(PDF)
