# Supplementary material for: Cardiac Alpha-Myosin (MYH6) Is the Predominant Sarcomeric Disease Gene for Familial Atrial Septal Defects
Source: PLoS One. 2011 Dec 14;6(12):e28872. doi: 10.1371/journal.pone.0028872 (PMC3237499; doi:10.1371/journal.pone.0028872)
Supplement: Data S1 — Gene variants identified in 31 index patients with familial ASDII. (PDF) [file pone.0028872.s001.pdf]

Supporting data 1

Genetic variants found in 31 ASDII patients of Caucasian origin

gene name	nucleotide change	amino acid change	web reference	number of affected individuals (Caucasian origin)	novel or putative disease associated variants	disease associated in pedigree	Allele frequencies according to EVS European / African
ACTC1	c.-23+53C>T			1	no		
ACTC1	c.808+76G>C		rs3729755	12	no		
ACTC1	c.990+64C>T		rs2307494	1	no		
CAV3	c.-67-117G>T		rs2072582	12	no		
CAV3	c.1-106G>A			18	no		
CAV3	c.27C>T	p.L9L	rs1974763	8	no		
CAV3	c.99C>T	p.N33N	rs1008642	19	no		
CAV3	c.114+26G>A			1	no		
CAV3	c.114+155C>A			1	no		
CAV3	c.114+171C>G		rs1558991	10	no		
CAV3	c.115-151A>G		rs6777678	10	no		
CAV3	c.115-89G>T		rs13060135	8	no		
CAV3	c.115-23G>C		rs57159780	3	no		
CAV3	c.123T>C	p.F41F		11	no		
CAV3	c.*36G>C			1	no		
CRYAB	c.165G>A	p.L55L	rs2228387	2	no		
CRYAB	c.324+4T>G		rs11603779	16	no		
CSRP3	c.150G>A	p.A50A	rs7124801	1	no		
CSRP3	c.213C>T	p.I71I	rs45476991	2	no		
CSRP3	c.336G>A	p.A112A	rs13451	3	no		
GLA	c.-12G>A		rs3027585	3	no		
GLA	c.-10C>T		rs2071225	4	no		
GLA	c.51C>T	p.R17R		1	no		
GLA	c.427G>A	p.A143T		1	no		
GLA	c.639+68A>G		rs3027589	3	no		
GLA	c.640-16A>G		rs2071397	6	no		
GLA	c.1000-22C>T		rs2071228	8	no		
GLA	c.1308+61T>G			1	no		
GLA	c.1308+84T>C			1	no		
GLA	c.1308+93G>A			1	no		
GLA	c.1308+136G>A		rs35019768	5	no		
GLA	c.1308+138A>C			1	no		
MYBPC3	c.472G>A	p.V158M	rs3729986	1	no		
MYBPC3	c.706A>G	p.S236G	rs3729989	7	no		
MYBPC3	c.772+28G>T			1	no		
MYBPC3	c.786C>T	p.T262T	rs11570058	7	no		
MYBPC3	c.1091-24C>T		rs2856650	6	no		
MYBPC3	c.1223+29G>A		rs11570078	7	no		
MYBPC3	c.1226+49T>C		rs896818	12	no		
MYBPC3	c.1608T>A	p.A536A		1	no		
MYBPC3	c.1897+24G>T			1	no		
MYBPC3	c.2308+18C>G		rs3729948	1	no		
MYBPC3	c.2497G>A	p.A833T		2	**yes**	no	A=6/2174 A= 0/1292
MYBPC3	c.2547C>T	p.V849V	rs3729953	1	no		
MYBPC3	c.2737+12C>T		rs3729936	1	no		
MYBPC3	c.3191-21A>G		rs11570115	2	no		
MYBPC3	c.3288G>A	p.E1096E	rs1052373	7	no		
MYBPC3	c.3627+49C>T		rs3729802	3	no		
MYBPC3	c.3815-66C>T		rs2290146	3	no		
MYH6	c.-75-1T>C			1	**yes**	no	A=0/2692 A= 0/2168
MYH6	c.50G>A	p.R17H		1	**yes**	**yes**	A=0/2692 A= 0/2168
MYH6	c.166G>A	p.G56R	rs28711516	6	no		
MYH6	c.201+16C>T		rs7147244	1	no		
MYH6	c.346-121C>G			2	no		
MYH6	c.393G>A	p.L131L	rs17277970	6	no		
MYH6	c.411G>A	p.E137E	rs2277474	12	no		
MYH6	c.427C>A	p.R143R	rs2277473	7	no		
MYH6	c.531-86A>G		rs440466	10	no		
MYH6	c.735+159T>C		rs12889823	1	no		
MYH6	c.736-105C>T		rs388914	12	no		
MYH6	c.736-69T>G			3	no		
MYH6	c.736-34G>A		rs11850295	1	no		
MYH6	c.799+122G>A			3	no		
MYH6	c.800-11A>G		rs434273	25	no		
MYH6	c.898+175G>A		rs17091631	6	no		
MYH6	c.999C>T	p.T333T		2	no		
MYH6	c.1003-29C>A		rs45466392	1	no		
MYH6	c.1335C>T	p.N445N		2	no		
MYH6	c.1410+112T>C		rs28671012	7	no		
MYH6	c.1582-39C>T		rs439735	11	no		
MYH6	c.1615T>C	p.C539R		1	**yes**	**yes**	C=0/2694 / C= 0/2170
MYH6	c.1628A>G	p.K543R		1	**yes**	**yes**	G=0/2694 / G=0/2170
MYH6	c.1891+170C>T		rs17091535	2	no		
MYH6	c.1892-103C>G		rs28730775	2	no		
MYH6	c.1962+39T>C		rs412768	16	no		
MYH6	c.1963-127C>T		rs17256246	3	no		
MYH6	c.2151C>T	p.Y717Y		3	no		
MYH6	c.2168+26C>G			3	no		
MYH6	c.2292+18C>T		rs452036	18	no		
MYH6	c.2686-35T>C		rs28730773	3	no		
MYH6	c.2946G>A	p.E982E		2	no		
MYH6	c.3010G>T	p.A1004S		1	**yes**	**yes**	T=3/2699 / T=0/2176
MYH6	c.3106-108T>C		rs17091453	2	no		
MYH6	c.3302T>C	p.V1101A	rs365990	18	no		
MYH6	c.3343-59G>A			1	no		
MYH6	c.3388G>A	p.A1130T	rs28730771	10	no		
MYH6	c.3480C>T	p.S1160S		1	no		
MYH6	c.3573C>T	p.A1191A		1	no		
MYH6	c.3609C>G	p.A1203A		1	**yes**	no	G=0/2292 / G=35/1919
MYH6	c.3627C>T	p.I1209I		1	**yes**	no	T=0/2554 / T=45/2081
MYH6	c.3733-78C>A			1	no		
MYH6	c.3978+166T>C		rs73604577	1	no		
MYH6	c.4011G>A	p.S1337S	rs451794	4	no		
MYH6	c.4136C>T	p.T1379M		1	no		
MYH6	c.4359+13C>T		rs8022522	13	no		
MYH6	c.4526-34T>C		rs178642	18	no		
MYH6	c.4650+24C>T		rs45536340	4	no		
MYH6	c.4651-17G>A		rs2071634	1	no		
MYH6	c.4914T>C	p.A1638A	rs178640	11	no		
MYH6	c.4959+46G>C		rs396024	4	no		
MYH6	c.4960-86G>A		rs45520434	4	no		
MYH6	c.4980C>T	p.D1660D	rs382872	4	no		
MYH6	c.5164-102T>A		rs178639	17	no		
MYH6	c.5164-74T>C		rs178638	16	no		
MYH6	c.5164-22A>G		rs178637	2	no		
MYH6	c.5259C>T	p.A1753A	rs8004990	3	no		
MYH6	c.5565+22A>G		rs8006357	14	no		
MYH6	c.5566-122A>C		rs35182223	14	no		
MYH6	c.5662-111G>T		rs10135780	6	no		
MYH6	c.5662-103T>C		rs178636	1	no		
MYH6	c.5797-128G>A		rs453361	4	no		

MYH7	c.-120-387G>A			1	no
MYH7	c.-120-198A>T			1	no
MYH7	c.-36C>T		rs45497293	2	no
MYH7	c.-9+23T>C		rs2239578	10	no
MYH7	c.-8-25G>T		rs3729992	2	no
MYH7	c.189C>T	p.T63T	rs2069540	10	no
MYH7	c.597A>G	p.A199A	rs2069541	1	no
MYH7	c.732C>T	p.F244F	rs2069542	5	no
MYH7	c.975C>T	p.D325D	rs2231124	2	no
MYH7	c.999+44T>C		rs3729810	2	no
MYH7	c.1062C>T	p.G354G	rs735712	2	no
MYH7	c.1095G>A	p.K365K	rs735711	4	no
MYH7	c.1128C>T	p.D376D	rs2231126	1	no
MYH7	c.2163-56A>G		rs3729818	3	no
MYH7	c.2424-58G>A		rs45571436	2	no
MYH7	c.2967T>C	p.I989I	rs7157716	8	no
MYH7	c.3853+21C>T		rs45584435	1	no
MYH7	c.3853+27T>A		rs2277475	5	no
MYH7	c.3972+62A>C		rs28730782	3	no
MYH7	c.3973-75G>A		rs45550632	2	no
MYH7	c.3973-30A>G		rs7159367	5	no
MYH7	c.3973-28C>T			1	no
MYH7	c.4520-63G>A		rs3729825	4	no
MYH7	c.4644+80C>T		rs3729828	5	no
MYH7	c.5106G>A	p.A1702A	rs3729830	2	no
MYH7	c.5284-45G>T		rs45468101	2	no
MYH7	c.5559+67A>T		rs3729832	2	no
MYH7	c.5655+21C>T		rs3729499	1	no
MYH7	c.5655+32G>A		rs3729833	4	no
MYH7	c.5791-64A>G		rs2284651	7	no
MYH7	c.5924+27C>A		rs12147570	4	no
MYH7	c.*113G>A		rs17794387	2	no
MYL2	c.4-77A>T		rs10849917	3	no
MYL2	c.132T>C	p.I44I	rs2301610	3	no
MYL2	c.274+27C>A			1	no
MYL2	c.274+53G>A		rs11065770	2	no
MYL2	c.275-58G>A		rs2071629	3	no
MYL2	c.353+51C>T		rs2233260	5	no
MYL2	c.756+76G>C		rs12812271	8	no
MYL3	c.129+44G>T		rs936175	6	no
MYL3	c.307+37A>C		rs2227294	6	no
MYL3	c.*89G>A			5	no
PRKAG2	c.-90G>T			1	no
PRKAG2	c.-26C>T			3	no
PRKAG2	c.114+12C>T			5	no
PRKAG2	c.114+43C>T			1	no
PRKAG2	c.186+111T>C		rs12671980	23	no
PRKAG2	c.187-96G>A		rs12375159	7	no
PRKAG2	c.187-85A>G		rs12374732	12	no
PRKAG2	c.466+51T>C			8	no
PRKAG2	c.467-172C>T			1	no
PRKAG2	c.754+141G>T			1	no
PRKAG2	c.865-151A>G		rs3762014	8	no
PRKAG2	c.1006-168A>G			4	no
PRKAG2	c.1006-121G>A			4	no
PRKAG2	c.1006-95A>T		rs17134620	5	no
PRKAG2	c.1006-83C>A			1	no
PRKAG2	c.1052-86C>T		rs2302530	10	no
PRKAG2	c.1052-42C>T		rs2302531	5	no
PRKAG2	c.1233+88G>A		rs2241053	8	no
PRKAG2	c.1437+47A>G			1	no
PRKAG2	c.1437+127T>G		rs2302532	7	no
PRKAG2	c.1438-80G>C			1	no
PRKAG2	c.*112A>G		rs8961	23	no
PRKAG2	c.*301A>G			1	no
TCAP	c.316C>T	p.R106C	rs45578741	1	no
TCAP	c.453A>C	p.A151A	rs1053651	17	no
TCAP	c.950+7C>G		rs45593534	1	no
TCAP	c.*199G>T		rs45503594	3	no
TNNC1	c.-27-144G>A		rs1035002	29	no
TNNI3	c.-98C>A		rs12973773	2	no
TNNI3	c.25-8T>A		rs3729836	12	no
TNNI3	c.108+21G>A		rs3729837	6	no
TNNI3	c.204G>T	p.R68R	rs3729711	6	no
TNNI3	c.373-10T>G		rs7252610	25	no
TNNI3	c.537G>A	p.E179E	rs3729841	7	no
TNNT2	c.-72-240C>T		rs45529540	2	no
TNNT2	c.134-50G>A		rs3729843	6	no
TNNT2	c.203+67G>A		rs1573230	12	no
TNNT2	c.207G>A	p.S69S	rs3729845	2	no
TNNT2	c.318C>T	p.I106I	rs3729547	10	no
TNNT2	c.571-32A>C		rs1104859	10	no
TNNT2	c.579+68C>T			1	no
TNNT2	c.690-86T>C		rs2275861	5	no
TNNT2	c.781-33C>T		rs2275863	2	no
TNNT2	c.*71C>T		rs3730244	1	no
TPM1	c.241-88G>A			20	no
TPM1	c.241-52T>A		rs28485524	6	no
TPM1	c.375-75A>G		rs4775614	27	no
TPM1	c.453C>A	p.A151A		27	no
TPM1	c.486T>C	p.Y162Y	rs11558747	2	no
TPM1	c.639+22G>C		rs28730802	1	no
TPM1	c.773-82G>A			6	no
TPM1	c.*148G>T		rs7668	2	no
