# Supplementary material for: Cardiac Alpha-Myosin (MYH6) Is the Predominant Sarcomeric Disease Gene for Familial Atrial Septal Defects
Source: PLoS One. 2011 Dec 14;6(12):e28872. doi: 10.1371/journal.pone.0028872 (PMC3237499; doi:10.1371/journal.pone.0028872)
Supplement: Data S2 — Fragments of a multiple sequence alignment of all 37 human myosin heavy chains with four homologs of known structure. Sequence identifiers and other sequence information are available in Supporting data 1. The yellow and green blocks represent alpha-helix and beta-sheet in solved structures. (A) Region around R17. (B) Region around C539 and K543. (C) Region around A1004. (PDF) [file pone.0028872.s002.pdf]

Supporting data 2

A

R17H

```
                 1   5    10   15   20   25   30
                 |...|....|....|....|....|....|..
myh6             MTDAQMADFGAAAQYLRKSEKERLEAQTRPFD   Class II
myh6_c/2mys      SPDAEMAAFGEAAPYLRKSEKERIEAQNKPFD.
myh15            LIKMDLSDLGEAAAFLRRSEAELLLLQATALD
myh7b            TAMMDVSELGESARYLRQGYQEMTKVHTIPWD
myh7             MGDSEMAVFGAAAPYLRKSEKERLEAQTRPFD
myh13            SSDAEMAIFGEAAPYLRKPEKERIEAQNRPFD
myh3             SSDTEMEVFGIAAPFLRKSEKERIEAQNQPFD.
myh8             SSDAEMAVFGEAAPYLRKSEKERIEAQNKPFD.
myh4             SSDSEMAIFGEAAPFLRKSEKERIEAQNKPFD.
myh1             SSDSEMAIFGEAAPFLRKSERERIEAQNKPFD.
myh2             SSDSELAVFGEAAPFLRKSERERIEAQNRPFD.
myh14            VPEAAQPFLFTPRGPSAGGGPGSGTSPQVEWT.
myh11            -MAQKGQLSDDEKFLFVDKNFINSPVAQADWA
myh11_c/1br2     --MSQKPLSDDEKFLFVDKNFVNNPLAQADWS.
myh10            -MAQRTGLEDPERYLFVDRAVIYNPATQADWT.
myh9             -----MAQQAADKYLYVDKNFINNPLAQADWA
myo5c            ---------MAVAELYTQYNRVWIPDPEEVWK   Class V
myo5b            ---------MSVGELYSQCTRVWIPDPDEVWR
myo5a_c/1oe9     ---------MAASELYTKYARVWIPDPEEVWK
myo5a            ---------MAASELYTKFARVWIPDPEEVWK
myo9a            HTLRIYPGAISEGTIYCPIPARKNSTAAEVIE   Class IX
myo9b            HLHIYPQLSTTESQASCRVTATKDSTTSDVIK
myo6             ---------------MEDGKPVWAPH---PTD   Class VI
myo6_p/2bkh      ---------------MEDGKPVWAPH---PTD
myo7a            ------------MVILQQGDHVWMDLRLGQEF   Class VII
myo7b            ------------MSGFRLGDHVWLEPPSTHKT
myo10            -----------MDNFFTEGTRVWLRE------   Class X
```

B

C539R K543R

```
                 530  535  540      545  550  555
                 --|....|....|----...-.|....|....|...
myh6             --GIMSILEEECM----FPK-ATDMTFKAKLYDNHL   Class II
myh6_c/2mys      --GIFSILEEECM----FPK-ATDTSFKNKLYDQHL
myh15            --GILSILEEECM----FPK-ATDLTFKTKLFDNHF
myh7b            --GILSILEEECM----FPK-ASDASFRAKLYDNHA
myh7             --GIMSILEEECM----FPK-ATDMTFKAKLFDNHL
myh13            --GIFSILEEECM----FPK-ATDTSFKNKLYDQHL
myh3             --GIFSILEEECM----FPK-ATDTSFKNKLYDQHL
myh8             --GIFSILEEECM----FPK-ATDTSFKNKLYDQHL
myh4             --GIFSILEEECM----FPK-ATDTSFKNKLYEQHL
myh1             --GIFSILEEECM----FPK-ATDTSFKNKLYEQHL
myh2             --GIFSILEEECM----FPK-ATDTSFKNKLYDQHL
myh14            PPGLLALLDEECW----FPK-ATDKSFVEKVAQEQ-
myh11            PPGVLALLDEECW----FPK-ATDKSFVEKLCTEQ-
myh11_c/1br2     PPGVLALLDEECW----FPK-ATDTSFVEKLIQEQ-
myh10            PPGVLALLDEECW----FPK-ATDKTFVEKLVQEQ-
myh9             PPGILALLDEECW----FPK-ATDKSFVEKVMQEQ-
myo1e            PPGIMSILDDVCATMHAVGE-GADQTLLQKLQMQI-   Class I
myo1f            PPGIMSVLDDVCATMHATGG-GADQTLLQKLQAAV-
myo1d            --GIIAILDDACM----NVGKVTDEMFLEALNSKL-
myo1g            --GILAVLDEACS----SAGTITDRIFLQTLDMHH-
myo1c            --GIISILDEECL----RPGEATDLTFLEKLEDTV-
myo1h            --GIISILDEECI----RPGPATDLSFLEKLEEKV-
myo1a            --GILAMLDEECL----RPGVVSDSTFLAKLNQLF-
myo1b            --GILAMLDEECL----RPGTVTDETFLEKLNQVC-
myo5c            --GILELLDEECL----LPH-GTDENWLQKLYNNFV   Class V
myo5b            --GILDLLDEECK----VPK-GTDQNWAQKLYDRH-
myo5a_c/1oe9     --GVLDLLDEECK----MPK-GSDDTWAQKLYNTHL
myo5a            --GILDLLDEECK----MPK-GTDDTWAQKLYNTHL
myo15a           --GILRILDDQCC----FPQ-ATDHTFLQKCHYHH-   Class XV
myo9a            --GLLHLLDEESN----FPQ-ATNQTLLDKFKHQH-   Class IX
myo9b            --GLFYLLDEESN----FPH-ATSQTLLAKFKQQH-
myo6             --GILDILDEENR----LPQ-PSDQHFTSAVHQKH-   Class VI
myo6_p/2bkh      --GILDILDEENR----LPQ-PSDQHFTSAGHQKH-
myo7a            --NIISLIDEESK----FPK-GTDTTMLHKLNSQH-   Class VII
myo7b            --SIISLLDEESR----FPQ-GTDLTMLQKLNSVH-
myo10            --GLLALINEESH----FPQ-ATDSTLLEKLHSQH-   Class X
myo3a            --GLLSLLDEESR----FPK-ATDQTLVEKFEGNL-   Class III
myo3b            --GLLALLDEESR----FPQ-ATDQTLVDKFEDNL-
myo18a           ARGLLWLLEEEAL----VPG-ASEDTLLERLFSYY-   Class XVIII
myo18b           ARGLFWVLDEEVH----VEG-SSDSVVLERLCAAFE
```

C

A1004S

```
                 995 1000 1005 1010
                 |....|....|....|.
myh6             TKEKKALQEAHQQALDD   Class II
myh6_c/2mys      TKEKKALQEAHQQTLDD
myh15            NRAAKVVQEAHQQTLDD
myh7b            TKEKKALQEAHQQALGD
myh7             TKEKKALQEAHQQALDD
myh13            TKEKKSLQEAHQQTLDD
myh3             TREKKALQEAHQQALDD
myh8             SKEKKALQETHQQTLDD
myh4             TKEKKALQEAHQQTLDD
myh1             TKEKKALQEAHQQTLDD
myh2             TKEKKALQEAHQQTLDD
myh14            SKERKLLEDRLAEFSSQ
myh11            SKERKLLEERISDLTTN
myh11_c/1br2TKERKLLEERVSDLTTN
myh10            IKEKKLMEDRIAECSSQ
myh9             AKEKKLLEDRIAEFTTN
```
