# Supplementary material for: Cardiac Alpha-Myosin (MYH6) Is the Predominant Sarcomeric Disease Gene for Familial Atrial Septal Defects
Source: PLoS One. 2011 Dec 14;6(12):e28872. doi: 10.1371/journal.pone.0028872 (PMC3237499; doi:10.1371/journal.pone.0028872)
Supplement: Data S3 — Database record identifiers and annotations of the 37 human genes for myosin heavy chains and of four homologs of solved structure. (PDF) [file pone.0028872.s003.pdf]

Supporting data 3

Gene name	Species	description	class	Gene id	Homologene	protein	SwissProt	Myosin_head	switch I region	switch II region	Motor_domain
MYH1	Homo sapiens	myosin, heavy chain 1, skeletal muscle, adult	class II	4619	117708	115527082		89..770	243..248	465..472	
MYH10	Homo sapiens	myosin, heavy chain 10, non-muscle	class II	4628	55941	41406064		87..771	238..243	460..467	
MYH11	Homo sapiens	myosin, heavy chain 11, smooth muscle	class II	4629	68258	92091586		87..778	245..250	467..474	
MYH11	Gallus gallus	myosin, heavy chain 11, smooth muscle	class II	396211	68258	45384060	MYH11_CHICK	86..777	244..249	466..473	
MYH13	Homo sapiens	myosin, heavy chain 13, skeletal muscle	class II	8735	55780	110624781		89..770	242..247	464..471	
MYH14	Homo sapiens	myosin, heavy chain 14, non-muscle	class II	79784	23480	116284396		108..796	266..271	487..494	
MYH15	Homo sapiens	myosin, heavy chain 15	class II	22989	18929	150010558		106..778	253..258	474..481	
MYH16	Homo sapiens	myosin, heavy chain 16 pseudogene									
MYH2	Homo sapiens	myosin, heavy chain 2, skeletal muscle, adult	class II	4620	75068	153791586		89..772	243..248	465..472	
MYH3	Homo sapiens	myosin, heavy chain 3, skeletal muscle, embryonic	class II	4621	20553	98986453		89..767	241..246	463..470	
MYH4	Homo sapiens	myosin, heavy chain 4, skeletal muscle	class II	4622	23019	110611903		89..770	243..248	465..472	
MYH6	Gallus gallus	myosin, heavy chain 6, cardiac muscle, alph	class II	427788	23019*	13432175	MYSS_CHICK	90..769	243..248		
**MYH6**	**Homo sapiens**	**myosin, heavy chain 6, cardiac muscle, alpha**	**class II**	**4624**	**124414**	**317373582**	**MYH6_HUMAN**	**88..768**	**241..246**	**463..470**	
MYH7	Homo sapiens	myosin, heavy chain 7, cardiac muscle, beta	class II	4625	68044	115496169	MYH7_HUMAN	88..766	240..245	462..469	
MYH7B	Homo sapiens	myosin, heavy chain 7B, cardiac muscle, beta	class II	57644	66117	114842389		128..815	285..290	507..514	
MYH8	Homo sapiens	myosin, heavy chain 8, skeletal muscle, perinatal	class II	4626	68256	153945790		91..769	243..248	465..472	
MYH9	Homo sapiens	myosin, heavy chain 9, non-muscle	class II	4627	68257	12667788		83..764	231..236	453..460	
MYO1A	Homo sapiens	myosin IA	class I	4640	21113	4885503		27..681	156..160	381..388	
MYO1B	Homo sapiens	myosin IB	class I	4430	7856	44889481		19..688	163..167	388..395	
MYO1C	Homo sapiens	myosin IC	class I	4641	32046	124494238		48..718	195..199	422..429	
MYO1D	Homo sapiens	myosin ID	class I	4642	45576	51100974		13..682	159..163	384..391	
MYO1E	Homo sapiens	myosin IE	class I	4643	55864	55956916		21..679	167..171	389..396	
MYO1F	Homo sapiens	myosin IF	class I	4542	56276	27544941		19..677	165..169	387..394	
MYO1G	Homo sapiens	myosin IG	class I	64005	27996	239582755		12..694	159..163	396..403	
MYO1H	Homo sapiens	myosin IH	class I	283446	82639	254028267		13..678	160..164	382..389	
MYO3A	Homo sapiens	myosin IIIA	class III	53904	49486	145275208		340..1041	484..488	718..725	<923..1052
MYO3B	Homo sapiens	myosin IIIB	class III	140469	51393	284172512		358..1056	489..493	723..730	
MYO5A	Gallus gallus	myosin VA (heavy chain 12, myoxin	class V	396237	20100	547967	MYO5A_CHICK	73..752		438..445	
MYO5A	Homo sapiens	myosin VA (heavy chain 12, myoxin)	class V	4644	20100	215982794		73..751	217..221	438..445	
MYO5B	Homo sapiens	myosin VB	class V	4645	49481	122937345		73..749	217..221	439..446	
MYO5C	Homo sapiens	myosin VC	class V	55930	68947	153945715		71..741	215..219	436..443	
MYO6	Homo sapiens	myosin VI	class VI	4646	56417	92859701		59..759	203..207	457..464	
MYO6	Sus scrofa	myosin VI	class VI	397085	nil	47522864	MYO6_PIG	59..760	203..207	458..465	
MYO7A	Homo sapiens	myosin VIIA	class VII	4647	219	189083798		67..729	210..214	438..445	
MYO7B	Homo sapiens	myosin VIIB	class VII	4648	81947	122937512		67..748			66..759
MYO9A	Homo sapiens	myosin IXA	class IX	4649	21371	156119615		149..683>			140..714
MYO9B	Homo sapiens	myosin IXB	class IX	4650	3058	33356170		147..666>			140..695
MYO10	Homo sapiens	myosin X	class X	4651	36328	154354979		65..725	218..222	435..442	
MYO15A	Homo sapiens	myosin XVA	class XV	51168	56504	118402590		1224..1887	1368..1372	1589..1596	
MYO18A	Homo sapiens	myosin XVIIIA	class XVIII	399687	7977	28416946		407..1173	553..557	796..803	
MYO18B	Homo sapiens	myosin XVIIIB	class XVIII	84700	53435	51317366		573..1321	714..718	952..959	
