# Supplementary figures and images for: Cardiac Alpha-Myosin (MYH6) Is the Predominant Sarcomeric Disease Gene for Familial Atrial Septal Defects
Source: PLoS One. 2011 Dec 14;6(12):e28872. doi: 10.1371/journal.pone.0028872 (PMC3237499; doi:10.1371/journal.pone.0028872)

Supplemental Figure 2

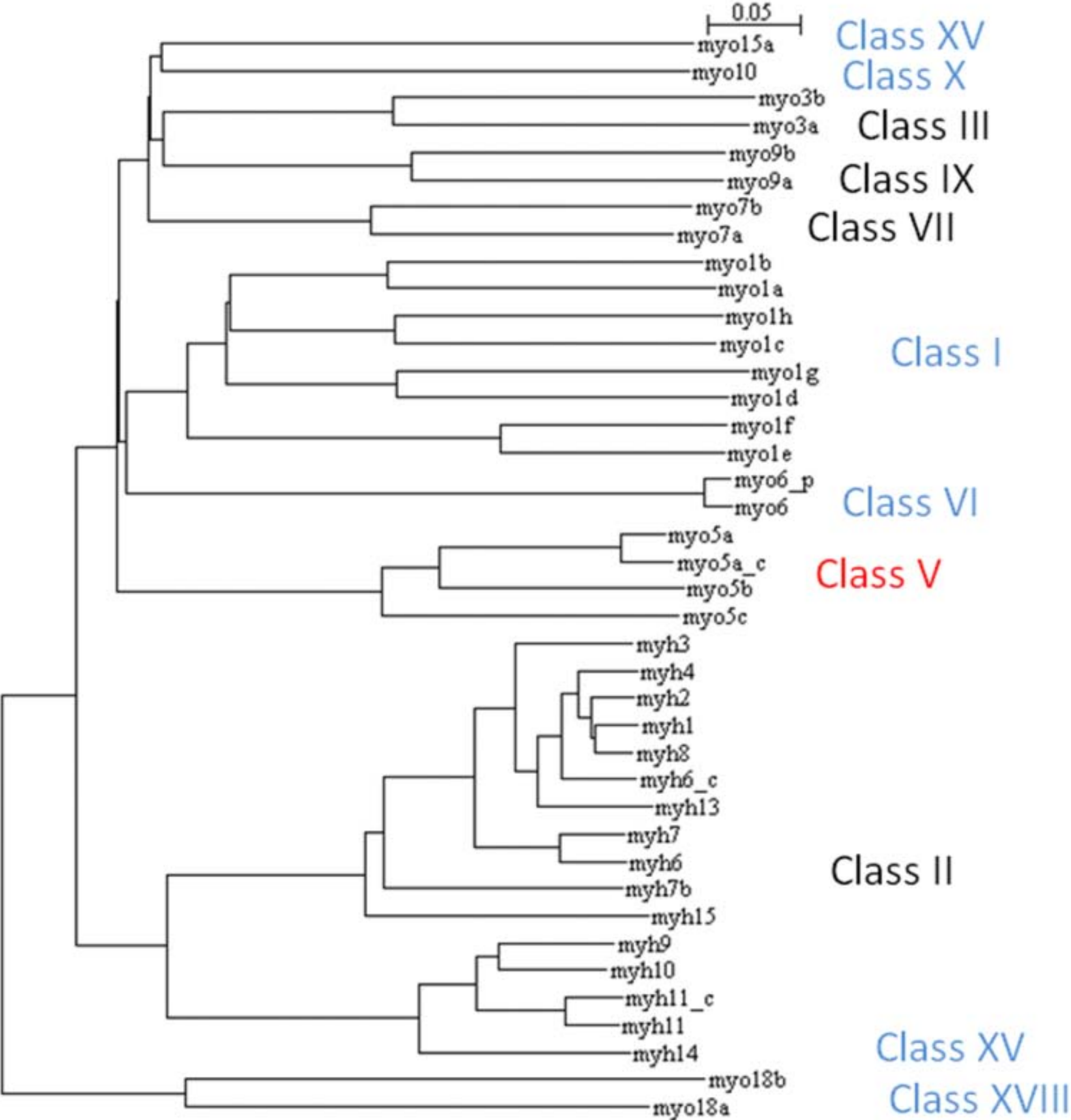

Supplement: Data S4 — Phylogenetic tree of the sequences aligned in the supporting data 1. Classes are indicated. (PDF) [file pone.0028872.s004.pdf]

## Supporting data 5

**A**

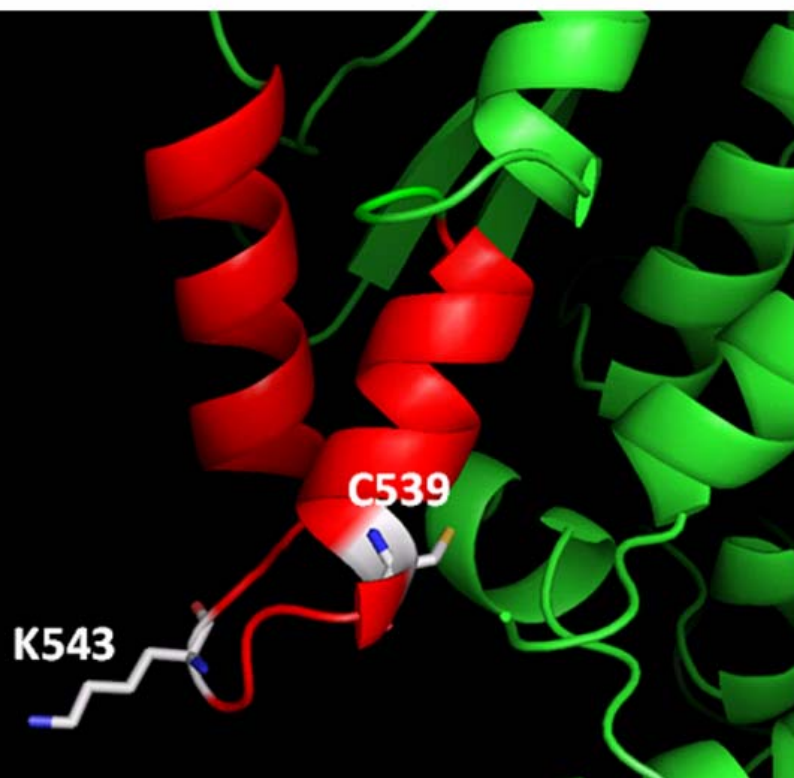

**B**

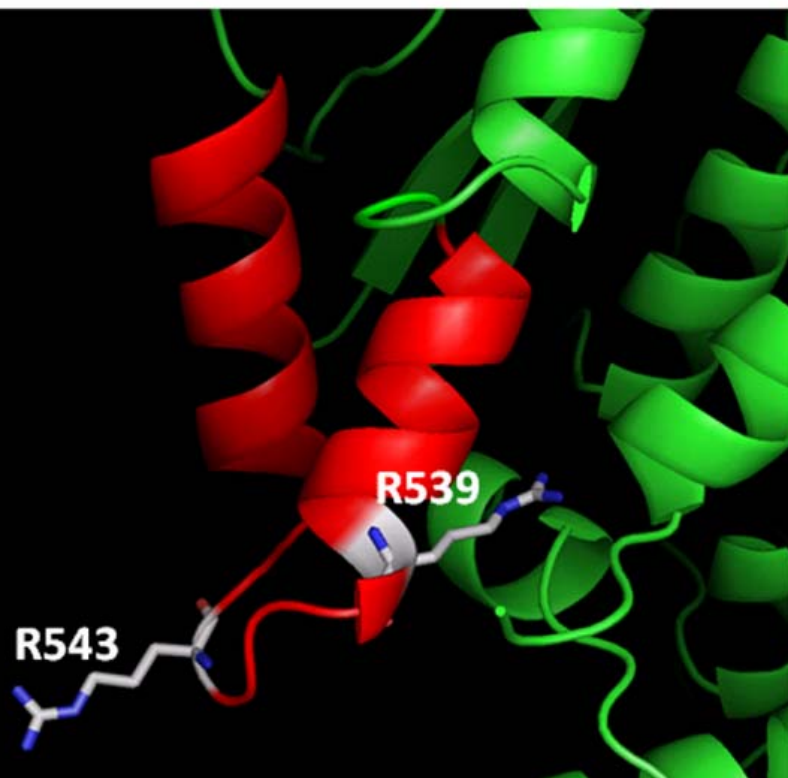

Supplement: Data S5 — Zoomed view of the structure from chicken myosin V (PDB:1OE9) [35]. The structure is displayed as a ribbon with the helix-loop-helix colored in red. (A) Original residues equivalent to human MYH6-K543 and MYH6-C539 are highlighted with side chains represented as sticks. These residues are conserved in the structure represented. (B) For illustration, mutants are represented by simple substitution of side chains without modifying the structure. R539 collides with the nearby helix since it is bulkier than the wild type residue. R543, also with a larger side chain than that of the wild type residue, points to the surface of interaction with actin according to a model by Holmes et al. [13]. This substitution may compromise the interaction between actin and myosin heavy chain. (PDF) [file pone.0028872.s005.pdf]
